# Statistical deferred weighted $\mathcal{B}$-summability and its applications to associated approximation theorems

**DOI:** 10.1186/s13660-018-1650-x

**Published:** 2018-03-27

**Authors:** T. Pradhan, S. K. Paikray, B. B. Jena, H. Dutta

**Affiliations:** 1 Department of Mathematics, Veer Surendra Sai University of Technology, Sambalpur, India; 2 Department of Mathematics, Gauhati University, Guwahati, India

**Keywords:** 40A05, 41A36, 40G15, Statistical convergence, Statistical deferred weighted $\mathcal{B}$-summability, Deferred weighted $\mathcal{B}$-statistical convergence, Positive linear operators, Rate of convergence, Banach space, Korovkin-type approximation theorems

## Abstract

The notion of statistical weighted $\mathcal{B}$-summability was introduced very recently (Kadak et al. in Appl. Math. Comput. 302:80–96, 2017). In the paper, we study the concept of statistical deferred weighted $\mathcal{B}$-summability and deferred weighted $\mathcal{B}$-statistical convergence and then establish an inclusion relation between them. In particular, based on our proposed methods, we establish a new Korovkin-type approximation theorem for the functions of two variables defined on a Banach space $C_{B}(\mathcal{D})$ and then present an illustrative example to show that our result is a non-trivial extension of some traditional and statistical versions of Korovkin-type approximation theorems which were demonstrated in the earlier works. Furthermore, we establish another result for the rate of deferred weighted $\mathcal{B}$-statistical convergence for the same set of functions via modulus of continuity. Finally, we consider a number of interesting special cases and illustrative examples in support of our findings of this paper.

## Introduction, preliminaries and motivation

In the interpretation of sequence spaces, the well-established traditional convergence has got innumerable applications where the convergence of a sequence demands that almost all elements are to assure the convergence condition, that is, every element of the sequence is required to be in some neighborhood of the limit. Nevertheless, there is such limitation in statistical convergence, where a set having a few elements that are not in the neighborhood of the limit is discarded. The preliminary idea of statistical convergence was presented and considered by Fast [2] and Steinhaus [3]. In the past few decades, statistical convergence has been an energetic area of research due essentially to the aspect that it is broader than customary (classical) convergence, and such hypothesis is talked about in the investigation in the fields of (for instance) Fourier analysis, functional analysis, number theory, and theory of approximation. In fact, see the current works [4–18], and [19] for detailed study.

Let the set of natural numbers be $\mathbb{N}$ and suppose that $K\subseteq \mathbb{N}$. Also, consider

$$K_{n}=\{k:k\leqq n\mbox{ and } k\in K\} $$

and suppose that $\vert K_{n} \vert $ is the cardinality of $K_{n}$. Then the natural density of *K* is defined by

$$d(K)=\lim_{n\rightarrow\infty}\frac{1}{n} \bigl\vert \{k:k\leqq n \mbox{ and } k\in K\} \bigr\vert $$

such that the limit exists.

A sequence $(x_{n})$ is statistically convergent (or stat-convergent) to *L* if, for every $\epsilon>0$,

$$K_{\epsilon}=\bigl\{ k:k\in \mathbb{N} \mbox{ and } \vert x_{k}-L \vert \geqq\epsilon\bigr\} $$

has zero natural (asymptotic) density (see [2, 3]). That is, for every $\epsilon>0$,

$$d(K_{\epsilon})=\lim_{n\rightarrow\infty}\frac{1}{n} \bigl\vert \bigl\{ k:k\leqq n\mbox{ and } \vert x_{k}-L \vert \geqq\epsilon \bigr\} \bigr\vert =0. $$

We write it as

$$\mathrm{stat}-\lim_{n\rightarrow\infty}x_{n}=L. $$

We present below an example to illustrate that every convergent sequence is statistically convergent but the converse is not true.

### Example 1

Let $x=(x_{n})$ be a sequence defined by

$$x_{n}= \textstyle\begin{cases} \frac{1}{5}& (n=m^{2}, m\in\mathbb{N})\\ \frac{n^{2}-1}{n^{2}+1} &(\textrm{otherwise}). \end{cases} $$

Here, the sequence $(x_{n})$ is statistically convergent to 1 even if it is not classically convergent.

In 2009, Karakaya and Chishti [20], introduced the fundamental concept of weighted statistical convergence, and later the definition was modified by Mursaleen *et al.* (see [21]).

Suppose that $(p_{k})$ is a sequence of non-negative numbers such that

$$P_{n}=\sum_{k=0}^{n}p_{k} \quad (p_{0}>0;n\rightarrow\infty). $$

Then, upon setting

$$t_{n}=\frac{1}{P_{n}}\sum_{k=0}^{n}p_{k}x_{k} \quad \bigl(n\in\mathbb{N}_{0}:=\mathbb{N}\cup\{0\}\bigr), $$

the given sequence $(x_{n})$ is weighted statistically convergent (or $\mathrm{stat}_{\bar{N}}$-convergent) to a number *L* if, for every $\epsilon>0$,

$$\bigl\{ k:k\leqq P_{n}\mbox{ and }p_{k} \vert x_{k}-L \vert \geqq\epsilon\bigr\} $$

has zero weighted density [21]. That is, for every $\epsilon>0$,

$$\lim_{n\rightarrow\infty}\frac{1}{P_{n}} \bigl\vert \bigl\{ k:k\leqq P_{n}\mbox{ and }p_{k} \vert x_{k}-L \vert \geqq\epsilon\bigr\} \bigr\vert =0. $$

Similarly, we say the sequence $(x_{n})$ is statistically weighted summable to *L* if, for every $\epsilon>0$,

$$\bigl\{ k:k\leqq n\mbox{ and } \vert t_{k}-L \vert \geqq\epsilon \bigr\} $$

has zero weighted summable density (see [21]). That is, for every $\epsilon>0$,

$$\lim_{n\rightarrow\infty}\frac{1}{n} \bigl\vert \bigl\{ k:k\leqq n \mbox{ and } \vert t_{k}-L \vert \geqq\epsilon\bigr\} \bigr\vert =0. $$

In the year 2013, Belen and Mohiuddine [5] established a new technique for weighted statistical convergence in terms of the de la Vallée Poussin mean, and it was subsequently investigated further by Braha *et al.* [8] as the $\Lambda_{n}$-weighted statistical convergence. Very recently, a certain class of weighted statistical convergence and associated Korovkin-type approximation theorems involving trigonometric functions have been introduced by Srivastava *et al.* (see, for details, [22]).

Suppose that *X* and *Y* are two sequence spaces, and let $\mathcal{A}=(a_{n,k})$ be a non-negative regular matrix. If for every $x_{k}\in X$ the series

$$\mathcal{A}_{n}x=\sum_{k=1}^{\infty}a_{n,k}x_{k} $$

converges for all $n\in\mathbb{N}$ and the sequence $(\mathcal{A}_{n}x)$ belongs to *Y*, then the matrix $\mathcal{A}:X\rightarrow Y$. Here, $(X,Y)$ denotes the set of all matrices that map *X* into *Y*.

Next, as regards the regularity condition, a matrix $\mathcal{A}$ is said to be regular if

$$\lim_{n\rightarrow\infty}\mathcal{A}_{n}x=L\quad \mbox{whenever } \lim_{k\rightarrow\infty}x_{k}=L. $$

We recall here that the well-known Silverman–Toeplitz theorem (see details in [23]) asserts that $\mathcal{A}=(a_{n,k})$ is regular if and only if the following conditions hold true:

(i) $\sup_{n\rightarrow\infty}\sum_{k=1}^{\infty} \vert a_{n,k} \vert <\infty$;
(ii) $\lim_{n\rightarrow\infty}a_{n,k}=0$ for each *k*;
(iii) $\lim_{n\rightarrow\infty}\sum_{k=1}^{\infty}a_{n,k}=1$.

Freedman and Sember [24] extended the definition of statistical convergence by considering the non-negative regular matrix $\mathcal{A}=(a_{n,k})$, which they called $\mathcal{A}$-statistical convergence. For any non-negative regular matrix $\mathcal{A}$, we say that a sequence $(x_{n})$ is said to be $\mathcal{A}$-statistically convergent (or $\mathrm{stat}_{\mathcal{A}}$-convergent) to a number *L* if, for each $\epsilon>0$,

$$d_{\mathcal{A}}(K_{\epsilon})=0, $$

where

$$K_{\epsilon}=\bigl\{ k:k\in\mathbb{N}\mbox{ and } \vert x_{k}-L \vert \geqq\epsilon\bigr\} . $$

We thus obtain that, for every $\epsilon>0$,

$$\lim_{n\rightarrow\infty}\sum_{k: \vert x_{k}-L \vert \geqq\epsilon}a_{n,k}=0. $$

In this case, we write

$$\mathrm{stat}_{\mathcal{A}}\lim x_{n}=L. $$

In the year 1998, the concept of $\mathcal{A}$-statistical convergence was extended by Kolk [25] to $\mathcal{B}$-statistical convergence with reference to $F_{\mathcal{B}}$-convergence (or $\mathcal{B}$-summable) due to Steiglitz (see [16]).

Suppose that $\mathcal{B}=(\mathcal{B}_{i})$ is a sequence of infinite matrices with $\mathcal{B}_{i}=(b_{n,k}(i))$. Then a sequence $(x_{n})$ is said to be $\mathcal{B}$-summable to the value $\mathcal{B}\lim_{n\rightarrow\infty}(x_{n})$ if

$$\lim_{n\rightarrow\infty}(\mathcal{B}_{i}x)_{n} =\lim _{n\rightarrow\infty}\sum_{k=0}^{\infty}b_{n,k}(i) (x)_{k}=\mathcal{B} \lim_{n\rightarrow\infty}(x_{n})\quad \mbox{uniformly for } i \quad \bigl(n,i\in\mathbb{N}_{0}:=\mathbb{N} \cup\{0\}\bigr). $$

The method $(\mathcal{B}_{i})$ is regular if and only if the following conditions hold true (see, for details, [26] and [27]):

(i) $\Vert \mathcal{B} \Vert = \sup_{n,i\rightarrow\infty}\sum_{k=0}^{\infty} \vert b_{n,k}(i) \vert <\infty$;
(ii) $\lim_{n\rightarrow\infty}b_{n,k}(i)=0$ uniformly in *i* for each $k\in\mathbb{N}$;
(iii) $\lim_{n\rightarrow\infty}\sum_{k=0}^{\infty}b_{n,k}(i)=1$ uniformly in *i*.

Let $K={k_{i}}\subset\mathbb{N}$ ($k_{i}< k_{i+1}$) for all *i*. The $\mathcal{B}$-density of *K* is defined by

$$d_{\mathcal{B}}(K)= \lim_{n\rightarrow\infty}\sum _{k=0}^{\infty}b_{n,k}(i) \quad \mbox{uniformly in }i, $$

provided the limit exists.

Let $\mathcal{R}^{+}$ be the set of all regular methods $\mathcal{B}$ with $b_{n,k}(i)\geqq0$ $(\forall n,k,i)$. Also, let $\mathcal{B}\in\mathcal{R}^{+}$. We say that a sequence $(x_{n})$ is $\mathcal{B}$-statistically convergent (or $\mathrm{stat}_{\mathcal{B}}$-convergent) to a number *L* if, for every $\epsilon>0$, we have

$$d_{\mathcal{B}}(K_{\epsilon})=0, $$

where

$$K_{\epsilon}=\bigl\{ k:k\in\mathbb{N}\mbox{ and } \vert x_{k}-L \vert \geqq\epsilon\bigr\} . $$

This implies that, for every $\epsilon>0$,

$$\lim_{n\rightarrow\infty}\sum_{k: \vert x_{k}-L \vert \geqq\epsilon}b_{n,k}(i)=0 \quad \mbox{uniformly in }i. $$

In this case, we write

$$\mathrm{stat}_{\mathcal{B}}\lim x_{n}=L. $$

Quite recently, Mohiuddine [28] introduced the notion of weighted $\mathcal{A}$-summability by using a weighted regular summability matrix. He also gave the definitions of statistical weighted $\mathcal{A}$-summability and weighted $\mathcal{A}$-statistical convergence. In particular, he proved a Korovkin-type approximation theorem under the consideration of statistically weighted $\mathcal{A}$-summable sequences of real or complex numbers. Subsequently, Kadak *et al.* [1] investigated the statistical weighted $\mathcal{B}$-summability by using a weighted regular matrix to establish some approximation theorems.

Motivated essentially by the above-mentioned works, here we present the (presumably new) notions of deferred weighted $\mathcal{B}$-statistical convergence and statistical deferred weighted $\mathcal{B}$-summability.

## Statistical deferred weighted $\mathcal{B}$-summability

In the present context, here we introduce the notions of deferred weighted $\mathcal{B}$-statistical convergence and statistical deferred weighted $\mathcal{B}$-summability by using the deferred weighted regular matrices (methods).

Let $(a_{n})$ and $(b_{n})$ be the sequences of non-negative integers fulfilling the conditions:

(i) $a_{n}< b_{n}$ ($n\in\mathbb{N}$)

and

(ii) $\lim_{n\rightarrow\infty}b_{n}=\infty$.

Conditions (i) and (ii) as above are the regularity conditions of the proposed deferred weighted mean [29].

Let $(p_{n})$ be the sequence of non-negative real numbers such that

$$P_{n}= \sum_{m=a_{n}+1}^{b_{n}}p_{m}. $$

In order to present the proposed deferred weighted mean $\sigma_{n}$, we first set

$$\sigma_{n}=\frac{1}{P_{n}} \sum_{m=a_{n}+1}^{b_{n}}p_{m}x_{m}. $$

The given sequence $(x_{n})$ is said to be deferred weighted summable (or $c^{D(\bar{N})}$-summable) to *L* if

$$\lim_{n\rightarrow\infty}\sigma_{n}=L. $$

In this case, we write

$$c^{D(\bar{N})}\lim_{n\rightarrow\infty}x_{n}=L. $$

We denote by $c^{D(\bar{N})}$ the set of all sequences that are deferred weighted summable.

Next, we present below the following definitions.

### Definition 1

A sequence $(x_{n})$ is said to be deferred weighted $\mathcal{B}$-summable (or $[D(\bar{N})_{\mathcal{B}};p_{n}]$-summable) to *L* if the $\mathcal{B}$-transform of $(x_{n})$ is deferred weighted summable to the same number *L*, that is,

$$\lim_{n\rightarrow\infty}\mathcal{B}_{n}^{(a_{n},b_{n})}(x) = \frac{1}{P_{n}}\sum_{m=a_{n}+1}^{b_{n}}\sum _{k=1}^{\infty}p_{m}b_{m,k}(i)x_{k}=L \quad \textrm{uniformly in }i. $$

In this case, we write

$$\bigl[D(\bar{N})_{\mathcal{B}};p_{n}\bigr]\lim_{n\rightarrow\infty}x_{n}=L. $$

We denote by $[D(\bar{N})_{\mathcal{B}};p_{n}]$ the set of all sequences that are deferred weighted $\mathcal{B}$-summable.

Definition 1 generalizes various known definitions as analyzed in Remark 1.

### Remark 1

If

$$a_{n}+1=\alpha(n)\quad \textrm{and}\quad b_{n}=\beta(n), $$

then the $\mathcal{B}_{n}^{(a_{n},b_{n})}(x)$ mean is close to the $\mathcal{B}_{n}^{a,b}(x_{n})$ mean [1], and if

$$a_{n}=0,\qquad b_{n}=n,\quad \textrm{and}\quad \mathcal{B}=A, $$

then the $\mathcal{B}_{n}^{(a_{n},b_{n})}(x)$ mean is the same as the $A_{m}^{\bar{N}}(x)$ mean [28]. Lastly, if

$$a_{n}=0,\qquad b_{n}=n,\quad \textrm{and}\quad \mathcal{B}=I \quad (\textrm{identity matrix}), $$

then the $\mathcal{B}_{n}^{(a_{n},b_{n})}(x)$ mean is the same as the weighted mean $(\bar{N},p_{n})$ [21].

### Definition 2

Let $\mathcal{B}=(b_{n,k}(i))$ and let $(a_{n})$ and $(b_{n})$ be sequences of non-negative integers. The matrix $\mathcal{B}=(\mathcal{B}_{i})$ is said to be a deferred weighted regular matrix (or deferred weighted regular method) if

$$\mathcal{B}x\in c^{D(\bar{N})}\quad (\forall x_{n}\in c) $$

with

$$c^{D(\bar{N})} \lim \mathcal{B}_{i}x_{n}=\mathcal{B}\lim (x_{n}) $$

and let it be denoted by $\mathcal{B}\in(c:c^{D(\bar{N})})$.

This means that $\mathcal{B}_{n}^{(a_{n},b_{n})}(x)$ exists for each $n\in \mathbb{N}$, $x_{n}\in c$ and

$$\lim_{n\rightarrow\infty}\mathcal{B}_{n}^{(a_{n},b_{n})}(x) \rightarrow L \quad \textrm{whenever }\lim_{n\rightarrow\infty}x_{n} \rightarrow L. $$

We denote by $\mathcal{R}^{+}_{D(w)}$ the set of all deferred weighted regular matrices (methods).

As a characterization of the deferred weighted regular methods, we present the following theorem.

### Theorem 1

*Let* $\mathcal{B}=(b_{n,k}(i))$ *be a sequence of infinite matrices*, *and let* $(a_{n})$ *and* $(b_{n})$ *be sequences of non*-*negative integers*. *Then* $\mathcal{B}\in (c:c^{D(\bar{N})} )$ *if and only if*

2.1 $$\begin{aligned} & \sup_{n}\sum_{k=1}^{\infty} \frac{1}{P_{n}} \Biggl\vert \sum_{m=a_{n}+1}^{b_{n}}p_{m}b_{m,k}(i) \Biggr\vert < \infty; \end{aligned}$$

2.2 $$\begin{aligned} &\lim_{n\rightarrow\infty}\frac{1}{P_{n}}\sum_{m=a_{n}+1}^{b_{n}}p_{m}b_{m,k}(i)=0 \quad \textrm{uniformly in } i\ (\textrm{for each }k,i\in\mathbb{N}) \end{aligned}$$

*and*

2.3 $$\begin{aligned} &\lim_{n\rightarrow\infty}\frac{1}{P_{n}}\sum_{m=a_{n}+1}^{b_{n}} \sum_{k=1}^{\infty}p_{m}b_{m,k}(i)=1 \quad \textrm{uniformly in }i. \end{aligned}$$

### Proof

Assume that (2.1)–(2.3) hold true and that $x_{n}\rightarrow L$ ($n\rightarrow\infty$). Then, for each $\epsilon>0$, there exists $m_{0}\in\mathbb{N}$ such that $\vert x_{n}-L \vert \leqq\epsilon$ ($m>m_{0}$). Thus, we have

$$\begin{aligned} \bigl\vert \mathcal{B}_{n}^{(a_{n},b_{n})}(x)-L \bigr\vert =& \Biggl\vert \frac{1}{P_{n}}\sum_{m=a_{n}+1}^{b_{n}} \sum_{k=1}^{\infty}p_{m}b_{m,k}(i)x_{k}-L \Biggr\vert \\ =& \Biggl\vert \frac{1}{P_{n}}\sum_{m=a_{n}+1}^{b_{n}} \sum_{k=1}^{\infty}p_{m}b_{m,k}(i) (x_{k}-L) +L \Biggl(\frac{1}{P_{n}}\sum _{m=a_{n}+1}^{b_{n}}\sum_{k=1}^{\infty}p_{m}b_{m,k}(i)-1 \Biggr) \Biggr\vert \\ \leqq& \Biggl\vert \frac{1}{P_{n}}\sum_{m=a_{n}+1}^{b_{n}} \sum_{k=1}^{\infty}p_{m}b_{m,k}(i) (x_{k}-L) \Biggr\vert \\ &{} + \vert L \vert \Biggl\vert \frac{1}{P_{n}}\sum_{m=a_{n}+1}^{b_{n}}\sum _{k=1}^{\infty}p_{m}b_{m,k}(i)-1 \Biggr\vert \\ \leqq& \Biggl\vert \frac{1}{P_{n}}\sum_{m=a_{n}+1}^{b_{n}} \sum_{k=1}^{b_{n-2}}p_{m}b_{m,k}(i) (x_{k}-L) \Biggr\vert \\ &{}+ \Biggl\vert \frac{1}{P_{n}}\sum_{m=a_{n}+1}^{b_{n}} \sum_{k=b_{n-1}}^{\infty}p_{m}b_{m,k}(i) (x_{k}-1) \Biggr\vert \\ &{}+ \vert L \vert \Biggl\vert \frac{1}{P_{n}}\sum _{m=a_{n}+1}^{b_{n}}\sum_{k=1}^{\infty}p_{m}b_{m,k}(i)-1 \Biggr\vert \\ \leqq&\sup_{k} \vert x_{k}-L \vert \sum _{k=1}^{b_{n-2}}\frac{1}{P_{n}}\sum _{m=a_{n}+1}^{b_{n}}p_{m}b_{m,k}(i)\\ &{} + \epsilon\frac{1}{P_{n}}\sum_{m=a_{n}+1}^{b_{n}}\sum _{k=1}^{\infty}p_{m}b_{m,k}(i) \\ &{}+ \vert L \vert \Biggl\vert \frac{1}{P_{n}}\sum _{m=a_{n}+1}^{b_{n}}\sum_{k=1}^{\infty}p_{m}b_{m,k}(i)-1 \Biggr\vert . \end{aligned}$$

Taking $n\rightarrow\infty$ and using (2.2) and (2.3), we get

$$\Biggl\vert \frac{1}{P_{n}}\sum_{m=a_{n}+1}^{b_{n}} \sum_{k=1}^{\infty}p_{m}a_{m,k}-L \Biggr\vert \leqq\epsilon, $$

which implies that

$$\lim_{n\rightarrow\infty} \frac{1}{P_{n}}\sum_{m=a_{n}+1}^{b_{n}} \sum_{k=1}^{\infty}p_{m}b_{m,k}(i)=L= \lim_{n\rightarrow\infty} (x_{n})\quad \textrm{uniformly in }i\ (i \geqq0), $$

since $\epsilon>0$ is arbitrary.

Conversely, let $\mathcal{B}\in(c:c^{D(\bar{N})})$ and $x_{n}\in c$. Then, since $\mathcal{B}x$ exists, we have the inclusion

$$\bigl(c:c^{D(\bar{N})}\bigr)\subset(c:L_{\infty}). $$

Clearly, there exists a constant *M* such that

$$\Biggl\vert \frac{1}{P_{n}}\sum_{m=a_{n}+1}^{b_{n}} \sum_{k=1}^{\infty}p_{m}b_{m,k}(i) \Biggr\vert \leqq M \quad (\forall m,n,i) $$

and the corresponding series

$$\Biggl\vert \frac{1}{P_{n}}\sum_{m=a_{n}+1}^{b_{n}} \sum_{k=1}^{\infty}p_{m}b_{m,k}(i) \Biggr\vert $$

converges uniformly in *i* for each *n*. Therefore, (2.1) is valid.

We now consider the sequence $x^{(n)}=(x_{k}^{(n)})\in c_{0}$ defined by

$$x_{k}^{(n)}= \textstyle\begin{cases} 1 &(n=k)\\ 0 &(n\neq k) \end{cases} $$

for all $n\in\mathbb{N}$ and $y=(y_{n})=(1,1,1,\ldots)\in c$. Then, since $\mathcal{B}x^{(n)}$ and $\mathcal{B}y$ belong to $c^{D(\bar{N})}$, thus (2.2) and (2.3) are fairly obvious. □

Next, for statistical version, we present below the following definitions.

### Definition 3

Let $\mathcal{B}\in\mathcal{R}^{+}_{D(w)}$, and let $(a_{n})$ and $(b_{n})$ be sequences of non-negative integers, and also let $K=(k_{i})\subset\mathbb{N}$ ($k_{i}\leq k_{i+1}$) for all *i*. Then the deferred weighted $\mathcal{B}$-density of *K* is defined by

$$d_{D(\bar{N})}^{\mathcal{B}}(K)=\lim_{n\rightarrow\infty}\frac{1}{S_{n}} \sum_{m=a_{n}+1}^{b_{n}} \sum _{k\in K}s_{m}b_{m,k}(i)\quad \textrm{uniformly in } i, $$

provided that the limit exists. A sequence $(x_{n})$ is said to be deferred weighted $\mathcal{B}$-statistically convergent to a number *L* if, for each $\epsilon>0$, we have

$$d_{D(\bar{N})}^{\mathcal{B}}(K_{\epsilon})=0\quad \textrm{uniformly in }i, $$

where

$$K_{\epsilon}=\bigl\{ k:k\in \mathbb{N} \textrm{ and } \vert x_{k}-L \vert \geqq\epsilon\bigr\} . $$

Here, we write

$$\mathrm{stat}_{D(\bar{N})}^{\mathcal{B}}\lim_{n\rightarrow\infty} (x_{n})=L. $$

### Definition 4

Let $\mathcal{B}\in\mathcal{R}^{+}_{D(w)}$, and let $(a_{n})$ and $(b_{n})$ be sequences of non-negative integers. We say that the sequence $(x_{n})$ is statistically deferred weighted $\mathcal{B}$-summable to a number *L* if, for each $\epsilon>0$, we have

$$d(E_{\epsilon})=0\quad \textrm{uniformly in }i, $$

where

$$E_{\epsilon}=\bigl\{ k:k\in \mathbb{N}\mbox{ and } \bigl\vert \mathcal{B}_{n}^{(a_{n},b_{n})}(x)-L \bigr\vert \geqq\epsilon\bigr\} . $$

Here, we write

$$\mathrm{stat}_{D(\bar{N})}\lim_{n\rightarrow\infty} (x_{n})=L\quad \Bigl(\mbox{or } \mathrm{stat}\lim_{n\rightarrow\infty}\mathcal{B}_{n}^{(a_{n},b_{n})}x=L \Bigr). $$

We now prove the following theorem which determines the inclusion relation between the deferred weighted $\mathcal{B}$-statistical convergence and the statistical deferred weighted $\mathcal{B}$-summability.

### Theorem 2

*Suppose that*

$$p_{n}b_{n,k}(i) \vert x_{n}-L \vert \leqq M \quad (n\in\mathbb{N};M>0). $$

*If a sequence* $(x_{n})$ *is deferred weighted* $\mathcal{B}$-*statistically convergent to a number* *L*, *then it is statistically deferred weighted* $\mathcal{B}$-*summable to the same number* *L*, *but the converse is not true*.

### Proof

Let

$$p_{n}b_{n,k}(i) \vert x_{n}-L \vert \leqq M \quad (n\in\mathbb{N};M>0). $$

Also let $(x_{n})$ be the deferred weighted $\mathcal{B}$-statistically convergent to *L*, we have

$$d_{D(\bar{N})}^{\mathcal{B}}(K_{\epsilon})=0\quad \textrm{uniformly in }i, $$

where

$$K_{\epsilon}=\bigl\{ k:k\in \mathbb{N}\mbox{ and } \vert x_{k}-L \vert \geqq\epsilon\bigr\} . $$

Thus we have

$$\begin{aligned} \bigl\vert \mathcal{B}_{n}^{(a_{n},b_{n})}(x_{n})-L \bigr\vert =& \Biggl\vert \frac{1}{P_{n}}\sum_{m=a_{n}+1}^{b_{n}} \sum_{k=1}^{\infty}p_{m}b_{m,k}(i) (x_{k}-L) \Biggr\vert \\ \leqq& \Biggl\vert \frac{1}{P_{n}}\sum_{m=a_{n}+1}^{b_{n}} \sum_{k=1}^{\infty}p_{m}b_{m,k}(i) (x_{k}-L ) \Biggr\vert + \vert L \vert \Biggl\vert \frac{1}{P_{n}}\sum_{m=a_{n}+1}^{b_{n}} \sum _{k=1}^{\infty}p_{m}b_{m,k}(i)-1 \Biggr\vert \\ \leqq& \Biggl\vert \frac{1}{P_{n}}\sum_{m=a_{n}+1}^{b_{n}} \sum_{k\in K_{\epsilon}}p_{m}b_{m,k}(i) (x_{k}-L ) \Biggr\vert \\ &{}+ \Biggl\vert \frac{1}{P_{n}}\sum_{m=a_{n}+1}^{b_{n}} \sum_{k\notin K_{\epsilon}}^{\infty}p_{m}b_{m,k}(i) (x_{k}-L ) \Biggr\vert + \Biggl\vert \frac{1}{P_{n}}\sum _{m=a_{n}+1}^{b_{n}} \sum_{k=1}^{\infty}p_{m}b_{m,k}(i)-1 \Biggr\vert \\ \leqq&\sup_{k\rightarrow\infty} \vert x_{k}-L \vert \frac{1}{P_{n}}\sum_{k\in K_{\epsilon}}\sum _{m=a_{n}+1}^{b_{n}}p_{m}b_{m,k}(i)+\epsilon \frac{1}{P_{n}}\sum_{m=a_{n}+1}^{b_{n}}\sum _{k\notin K_{\epsilon}}p_{m}b_{m,k}(i) \\ &{}+ \vert L \vert \Biggl\vert \frac{1}{P_{n}}\sum _{m=a_{n}+1}^{b_{n}}\sum_{k\in K_{\epsilon}}p_{m}b_{m,k}(i)-1 \Biggr\vert \rightarrow\epsilon\quad (n\rightarrow\infty), \end{aligned}$$

which implies that $\mathcal{B}_{n}^{(a_{n},b_{n})}(x_{n})\rightarrow L$ ($n\rightarrow\infty$). This implies that the sequence $(x_{n})$ is deferred weighted $\mathcal{B}$-summable to the number *L*, and hence the sequence $(x_{n})$ is statistically deferred weighted $\mathcal{B}$-summable to the same number *L*. □

In order to prove that the converse is not true, we present Example 2 (below).

### Example 2

Let us consider the infinite matrices $\mathcal{B}=(\mathcal{B}_{i})$ with $\mathcal{B}_{i}=(b_{n,k}(i))$ given by (see [1])

$$ x_{n}= \textstyle\begin{cases} \frac{1}{n+1}& (i\leqq k\leqq i+n) \\ 0&(\textrm{otherwise}). \end{cases} $$

We also suppose that $a_{n}=2n$, $b_{n}=4n$, and $p_{n}=1$. It can be easily seen that $\mathcal{B}\in\mathcal{R}_{w}^{+}$. We also consider the sequence $(x_{n})$ by

2.4 $$ x_{n}= \textstyle\begin{cases} 0& (n \textrm{ is even}) \\ 1& (n \textrm{ is odd}). \end{cases} $$

Since $P_{n}=2n$, we get

$$\frac{1}{P_{n}}\sum_{m=a_{n}+1}^{b_{n}}\sum _{k=1}^{\infty}p_{m}b_{m,k}(i)x_{k} =\frac{1}{2n}\sum_{m=2n+1}^{4n} \frac{1}{n+1}\sum_{k=i}^{i+n}x_{k}= \frac{1}{2n}\sum_{m=2n+1}^{4n} \frac{1}{2}=\frac{1}{2}. $$

Clearly, the sequence $(x_{n})$ is neither convergent nor statistically convergent and also the sequence $(x_{n})$ is not statistically weighted $\mathcal{B}$-summable and weighted $\mathcal{B}$-statistically convergent. However, the sequence $(x_{n})$ is deferred weighted $\mathcal{B}$-summable to $\frac{1}{2}$, so it is statistically deferred weighted $\mathcal{B}$-summable to $\frac{1}{2}$, but the sequence $(x_{n})$ is not deferred weighted $\mathcal{B}$-statistically convergent.

## A Korovkin-type theorem via statistical deferred weighted $\mathcal{B}$-summability

In the last few decades, many researchers emphasized expanding or generalizing the Korovkin-type hypotheses from numerous points of view in light of a few distinct angles, containing (for instance) space of functions, Banach spaces summability theory, etc. Certainly, the change of Korovkin-type hypothesis is far from being finished till today. For additional points of interest and outcomes associated with the Korovkin-type hypothesis and other related advancements, we allude the reader to the current works [7–10, 22], and [17]. The main objective of this paper is to extend the notion of statistical convergence by the help of the deferred weighted regular technique and to show how this technique leads to a number of results based upon an approximation of functions of two variables over the Banach space $C_{B}(\mathcal{D})$. Moreover, we establish some important approximation theorems related to the statistical deferred weighted $\mathcal{B}$-summability and deferred weighted $\mathcal{B}$-statistical convergence, which will effectively extend and improve most (if not all) of the existing results depending upon the choice of sequences of the deferred weighted $\mathcal{B}$ means. Based upon the proposed methodology and techniques, we intend to estimate the rate of convergence and investigate the Korovkin-type approximation results. In fact, we extend here the result of Kadak *et al.* [1] by using the notion of statistical deferred weighted $\mathcal{B}$-summability and present the following theorem.

Let $\mathcal{D}$ be any compact subset of the real two-dimensional space. We denote by $C_{B}(\mathcal{D})$ the space of all continuous real-valued functions on $\mathcal{D}=I\times I$ ($I=[0,A]$), $A\leq\frac{1}{2}$ and equipped with the norm

$$\Vert f \Vert _{C_{B}(\mathcal{D})} =\sup\bigl\{ \bigl\vert f(x,y) \bigr\vert :(x,y)\in\mathcal{D}\bigr\} ,\quad f\in C_{B}(\mathcal{D}). $$

Let $T:C_{B}(\mathcal{D})\rightarrow C_{B}(\mathcal{D})$ be a linear operator. Then we say that *T* is a positive linear operator provided

$$f\geqq0\quad \textrm{implies} \quad T(f)\geqq0. $$

Also, we use the notation $T(f;x,y)$ for the values of $T(f)$ at the point $(x,y)\in\mathcal{D}$.

### Theorem 3

*Let* $\mathcal{B}\in \mathcal{R}^{+}_{D(w)}$, *and let* $(a_{n})$ *and* $(b_{n})$ *be sequences of non*-*negative integers*. *Let* $T_{n}$ ($n\in \mathbb{N}$) *be a sequence of positive linear operators from* $C_{B}(\mathcal{D})$ *into itself*, *and let* $f\in C_{B}(\mathcal{D})$. *Then*

3.1 $$\begin{aligned} & \mathrm{stat}_{D(\bar{N})}\lim_{n} \bigl\Vert T_{n}\bigl(f(s,t);x,y\bigr)- f(x,y) \bigr\Vert _{C_{B}(\mathcal{D})}=0, \quad f\in C_{B}(\mathcal{D}) \end{aligned}$$

*if and only if*

3.2 $$\begin{aligned} & \mathrm{stat}_{D(\bar{N})}\lim_{n} \bigl\Vert T_{n}\bigl(f_{j}(s,t);x,y\bigr)- f(x,y) \bigr\Vert _{C_{B}(\mathcal{D})}=0, \quad (j=0,1,2,3), \end{aligned}$$

*where*

$$\begin{aligned} f_{0}(s,t)&=1, \qquad f_{1}(s,t)=\frac{s}{1-s}, \qquad f_{2}(s,t)=\frac{t}{1-t}\quad \textit{and} \\ f_{3}(s,t)&= \biggl(\frac{s}{1-s} \biggr)^{2}+ \biggl( \frac{t}{1-t} \biggr)^{2}. \end{aligned}$$

### Proof

Since each of the functions $f_{j}(s,t)\in C_{B}(\mathcal{D})$, the following implication

$$(3.1)\quad \Longrightarrow\quad (3.2) $$

is fairly obvious. In order to complete the proof of Theorem 3, we first assume that (3.2) holds true. Let $f\in C_{B}(\mathcal{D})$, $\forall(x,y)\in\mathcal{D}$. Since $f(x,y)$ is bounded on $\mathcal{D}$, then there exists a constant $\mathcal{M}>0$ such that

$$\bigl\vert f(x,y) \bigr\vert \leqq \mathcal{M}\quad (\forall x,y\in \mathcal{D}), $$

which implies that

3.3 $$ \bigl\vert f(s,t)-f(x,y) \bigr\vert \leqq 2\mathcal{M} \quad (s,t,x,y\in\mathcal{D}). $$

Clearly, *f* is a continuous function on $\mathcal{D}$, for given $\epsilon>0$, there exists $\delta=\delta(\epsilon)>0$ such that

3.4 $$ \bigl\vert f(s,t)-f(x,y) \bigr\vert < \epsilon\quad \mbox{whenever } \biggl\vert \frac{s}{1-s}-\frac{x}{1-x} \biggr\vert < \delta \mbox{ and } \biggl\vert \frac{t}{1-t}-\frac{y}{1-y} \biggr\vert < \delta $$

for all $s,t,x,y\in\mathcal{D}$.

From equations (3.3) and (3.4), we get

3.5 $$ \bigl\vert f(s,t)-f(x,y) \bigr\vert < \epsilon+ \frac{2\mathcal{M}}{\delta^{2}} \bigl(\bigl[\varphi(s,x)\bigr]^{2}+\bigl[\varphi(t,y) \bigr]^{2} \bigr), $$

where

$$\varphi(s,x)=\frac{s}{1-s}-\frac{x}{1-x}\quad \mbox{and}\quad \varphi(t,y)=\frac{t}{1-t}-\frac{y}{1-y}. $$

Since the function $f\in C_{B}(\mathcal{D})$, inequality (3.5) holds for $s,t,x,y\in\mathcal{D}$.

Now, since the operator $T_{n}(f(s,t);x,y)$ is linear and monotone, so inequality (3.5) under this operator becomes

3.6 $$\begin{aligned} \bigl\vert T_{n}\bigl(f(s,t);x,y\bigr)-f(x,y) \bigr\vert =& \bigl\vert T_{n}\bigl(f(s,t)-f(x,y);x,y\bigr)+f(x,y) \bigl[T_{k}(f_{0};x,y)-f_{0}\bigr] \bigr\vert \\ \leqq& \bigl\vert T_{n}\bigl(f(s,t)-f(x,y);x,y\bigr)+\mathcal{M} \bigl[T_{k}(1;x,y)-1\bigr] \bigr\vert \\ \leqq& \biggl\vert T_{n} \biggl(\epsilon+\frac{2\mathcal{M}}{\delta^{2}} \bigl[ \varphi(s,x)^{2}+\varphi(t,y)^{2} \bigr];x,y \biggr) \biggr\vert \\ &{} +\mathcal{M} \bigl\vert T_{n}(1;x,y)-1 \bigr\vert \\ \leqq&\epsilon+(\epsilon+\mathcal{M}) \bigl\vert T_{n}(f_{0};x,y)-f_{0}(x,y) \bigr\vert \\ &{} +\frac{2\mathcal{M}}{\delta^{2}} \bigl\vert T_{n}(f_{3};x,y)-f_{3}(x,y) \bigr\vert \\ &{}-\frac{4\mathcal{M}}{\delta^{2}} \biggl(\frac{x}{1-x} \biggr) \bigl\vert T_{n}(f_{1};x,y)-f_{1}(x,y) \bigr\vert \\ &{}-\frac{4\mathcal{M}}{\delta^{2}} \biggl(\frac{y}{1-y} \biggr) \bigl\vert T_{n}(f_{2};x,y)-f_{2}(x,y) \bigr\vert \\ &{}+\frac{2\mathcal{M}}{\delta^{2}} \biggl( \biggl(\frac{x}{1-x} \biggr)^{2} + \biggl(\frac{y}{1-y}^{2} \biggr) \biggr) \bigl\vert T_{n}(f_{0};x,y)-f_{0}(x,y) \bigr\vert \\ \leqq &\epsilon+ \biggl(\epsilon+\mathcal{M}+\frac{4\mathcal{M}}{\delta^{2}} \biggr) \bigl\vert T_{n}(1;x,y)-1 \bigr\vert \\ &{}+\frac{4\mathcal{M}}{\delta^{2}} \bigl\vert T_{n}(f_{1};x,y)-f_{1}(x,y) \bigr\vert +\frac{4\mathcal{M}}{\delta^{2}} \bigl\vert T_{n}(f_{2};x,y)-f_{2}(x,y) \bigr\vert \\ &{}+\frac{2\mathcal{M}}{\delta^{2}} \bigl\vert T_{n}(f_{3};x,y)-f_{3}(x,y) \bigr\vert . \end{aligned}$$

Next, taking $\sup_{x,y\in\mathcal{D}}$ on both sides of (3.6), we get

3.7 $$ \bigl\Vert T_{n}\bigl(f(s,t);x,y\bigr)-f(x,y) \bigr\Vert _{C_{B}(\mathcal{D})} \leqq\epsilon+ N\sum_{j=0}^{3} \bigl\Vert T_{n}\bigl(f_{j}(s,t);x,y\bigr)-f_{j}(x,y) \bigr\Vert _{C_{B}(\mathcal{D})}, $$

where

$$N= \biggl\{ \epsilon+\mathcal{M}+\frac{4\mathcal{M}}{\delta^{2}} \biggr\} . $$

We now replace $T_{n}(f(s,t);x,y)$ by

$$\mathfrak{L}_{n}\bigl(f(s,t);x,y\bigr)=\frac{1}{P_{n}}\sum _{m={a_{n}+1}}^{b_{n}} \sum_{k=0}^{\infty}p_{m}b_{m,k}(i)T_{k} \bigl(f(s,t);x,y\bigr)\quad (\forall i,m\in\mathbb{N}) $$

in equation (3.7).

Now, for given $r>0$, we choose $0<\epsilon'<r$, and by setting

$$\mathcal{K}_{n}= \bigl\vert \bigl\{ n:n\leqq \mathbb{N}\mbox{ and } \bigl\vert \mathfrak{L}_{n}\bigl(f(s,t);x,y\bigr)-f(x,y) \bigr\vert \geqq r\bigr\} \bigr\vert $$

and

$$\mathcal{K}_{j,n}= \biggl\vert \biggl\{ n:n\leqq \mathbb{N}\mbox{ and } \bigl\vert \mathfrak{L}_{n}\bigl(f_{j}(s,t);x,y \bigr)-f_{j}(x,y) \bigr\vert \geqq \frac{r-\epsilon'}{4N} \biggr\} \biggr\vert \quad (j=0,1,2,3), $$

we easily find from (3.7) that

$$\mathcal{K}_{n}\leqq \sum_{j=0}^{3} \mathcal{K}_{j,n}. $$

Thus, we have

3.8 $$ \frac{ \Vert \mathcal{K}_{n} \Vert _{C_{B}(\mathcal{D})}}{n}\leqq \sum_{j=0}^{3} \frac{ \Vert \mathcal{K}_{j,n} \Vert _{C_{B}(\mathcal{D})}}{n}. $$

Clearly, from the above supposition for the implication in (3.2) and Definition 4, the right-hand side of (3.8) tends to zero $(n\rightarrow\infty)$. Subsequently, we obtain

$$\mathrm{stat}_{D(\bar{N})}\lim_{n\rightarrow\infty} \bigl\Vert T_{n} \bigl(f_{j}(s,t);x,y\bigr)-f_{j}(x,y) \bigr\Vert _{C_{B}(\mathcal{D})}=0\quad (j=0,1,2,3). $$

Hence, implication (3.1) is fairly true, which completes the proof of Theorem 3. □

### Remark 2

If

$$\mathcal{B}=\mathcal{A},\qquad a_{n}=0,\quad \mbox{and}\quad b_{n}=n \quad (\forall n) $$

in our Theorem 3, then we obtain a statistical weighted $\mathcal{A}$-summability version of Korovkin-type approximation theorem (see [28]). Furthermore, if we substitute

$$a_{n}+1=\alpha(n)\quad \textrm{and}\quad b_{n}=\beta(n)\quad (\forall n) $$

in our Theorem 3, then we obtain a statistical weighted $\mathcal{B}$-summability version of Korovkin-type approximation theorem (see [1]). Finally,

$$\mathcal{B}=I\quad (\textrm{identity matrix}),\quad a_{n}=0,\quad \textrm{and}\quad b_{n}=n \quad (\forall n) $$

in our Theorem 3, then we obtain a statistical weighted convergence version of Korovkin-type approximation theorem (see [19]).

Now we recall the generating function type *Meyer–König and Zeller operators* of two variables (see [30] and [31]).

Let us take the following sequence of generalized linear positive operators:

3.9 $$\begin{aligned} L_{n,m}\bigl(f(s,t);x,y\bigr) =&\frac{1}{h_{n}(x,s)h_{m}(y,t)}\sum _{k=0}^{\infty}\sum_{l=0}^{\infty} f \biggl(\frac{a_{k,n}}{a_{k,n}+q_{n}},\frac{c_{l,m}}{c_{l,m}+r_{m}} \biggr) \\ &{}\times\Gamma_{k,n}(s) \Gamma_{l,m}(t)x^{k}y^{l}, \end{aligned}$$

where

$$0\leqq\frac{a_{k,n}}{a_{k,n}+q_{n}}\leqq A\quad \textrm{and}\quad 0\leqq\frac{c_{l,m}}{c_{l,m}+r_{m}} \leqq B\quad \bigl(\forall A,B\in(0,1)\bigr). $$

For the sequences of functions, $(\Gamma_{k,n}(s) )_{n\in\mathbb{N}}$ and $(\Gamma_{l,m}(t) )_{n\in\mathbb{N}}$ are the generating functions, $h_{n}(x,s)$ and $h_{m}(y,t)$ are defined by

$$h_{n}(x,s)=\sum_{k=0}^{\infty} \Gamma_{k,n}(s)x^{k}\quad \textrm{and}\quad h_{m}(y,t) =\sum_{l=0}^{\infty} \Gamma_{l,m}(t)y^{l}\quad \bigl(s,t\in I\times I\subset \mathbb{R}^{2}\bigr). $$

Because the nodes are given by

$$s=\frac{a_{k,n}}{a_{k,n}+q_{n}}\quad \textrm{and}\quad t=\frac{c_{l,m}}{c_{l,m}+r_{m}}, $$

the denominators of

$$\frac{s}{1-s}=\frac{a_{n,k}}{q_{n}}\quad \textrm{and}\quad \frac{t}{1-t}= \frac{c_{l,m}}{r_{m}} $$

are independent of *k* and *l*, respectively.

We also suppose that the following conditions hold true:

(i) $h_{n}(x,s)=(1-x)h_{n+1}(x,s)$ and $h_{m}(y,t)=(1-y)h_{m+1}(y,t)$;
(ii) $q_{n}\Gamma_{k,n+1}(s)=a_{k+1,n}\Gamma_{k+1,n}(s)$ and $r_{m}\Gamma_{l,m+1}(t)=c_{l+1,m}\Gamma_{l+1,m}(t)$;
(iii) $q_{n}\rightarrow\infty$, $\frac{q_{n+1}}{q_{n}}\rightarrow\infty$ ($r_{m}\rightarrow\infty$), $\frac{r_{m+1}}{r_{m}}\rightarrow\infty$ and $q_{n},r_{m}\neq0$ ($\forall m,n$);
(iv) $a_{k+1,n}-a_{k,n+1}=\phi_{n}$ and $c_{l+1,m}-a_{l,m+1}=\psi_{m}$,

where

$$\phi_{n}\leqq n\leqq\infty,\qquad \psi_{m}\leqq m\leqq \infty\quad \mbox{and}\quad a_{0,n}=c_{m,0}=0. $$

It is easy to see that $L_{n}(f(s,t);x,y)$ is positive linear operators. We also observe that

$$L_{n}(1;x,y)=1,\qquad L_{n} \biggl(\frac{s}{1-s};x,y \biggr)=\frac{x}{1-x},\qquad L_{n} \biggl(\frac{t}{1-t};x,y \biggr)=\frac{t}{1-t} $$

and

$$L_{n} \biggl( \biggl(\frac{s}{1-s} \biggr)^{2}+ \biggl( \frac{t}{1-t} \biggr)^{2};x,y \biggr) =\frac{x^{2}}{(1-x)^{2}} \frac{q_{n+1}}{q_{n}}+\frac{y^{2}}{(1-y)^{2}}\frac{r_{n+1}}{r_{n}} +\frac{x}{1-x} \frac{\phi_{n}}{q_{n}}+\frac{y}{1-y}\frac{\psi_{n}}{r_{n}}. $$

### Example 3

Let $T_{n}:C_{B}(\mathcal{D})\rightarrow C_{B}(\mathcal{D})$, $\mathcal{D}=[0,A]\times [0,A]$, $A\leq\frac{1}{2}$ be defined by

3.10 $$ T_{n}(f;x,y)=(1+x_{n})L_{n}(f;x,y), $$

where $(x_{n})$ is a sequence defined as in Example 2. It is clear that the sequence $(T_{n})$ satisfies the conditions (3.2) of our Theorem 3, thus we obtain

$$\begin{aligned} &\mathrm{stat}_{D(\bar{N})}\lim_{n} \bigl\Vert T_{n}(1;x,y)- 1 \bigr\Vert _{C_{B}(\mathcal{D})}=0 \\ &\mathrm{stat}_{D(\bar{N})}\lim_{n} \biggl\Vert T_{n} \biggl(\frac{s}{1-s};x,y \biggr)-\frac{x}{1-x} \biggr\Vert _{C_{B}(\mathcal{D})}=0 \\ &\mathrm{stat}_{D(\bar{N})}\lim_{n} \biggl\Vert T_{n} \biggl(\frac{t}{1-t};x,y \biggr)-\frac{y}{1-y} \biggr\Vert _{C_{B}(\mathcal{D})}=0 \end{aligned}$$

and

$$\mathrm{stat}_{D(\bar{N})}\lim_{n} \biggl\Vert T_{n} \biggl[ \biggl(\frac{s}{1-s} \biggr)^{2}+ \biggl( \frac{t}{1-t} \biggr)^{2};x,y \biggr]- \biggl[ \biggl( \frac{s}{1-s} \biggr)^{2}+ \biggl(\frac{t}{1-t} \biggr)^{2} \biggr] \biggr\Vert _{C_{B}(\mathcal{D})}=0. $$

Therefore, from Theorem 3, we have

$$\mathrm{stat}_{D(\bar{N})}\lim_{n} \bigl\Vert T_{n}\bigl(f(s,t);x,y\bigr)- f(x,y) \bigr\Vert _{C_{B}(\mathcal{D})}=0, \quad f\in C_{B}(\mathcal{D}). $$

However, since $(x_{n})$ is not statistically weighted $\mathcal{B}$-summable, so the result of Kadak *et al.* [1], p. 85, Theorem 3, certainly does not hold for the operators defined by us in (3.10). Moreover, as $(x_{n})$ is statistically deferred weighted $\mathcal{B}$-summable, therefore we conclude that our Theorem 3 works for the operators which we consider here.

## Rate of deferred weighted $\mathcal{B}$-statistical convergence

In this section, we compute the rate of deferred weighted $\mathcal{B}$-statistical convergence of a sequence of positive linear operators of functions of two variables defined on $C_{B}(\mathcal{D})$ into itself by the help of modulus of continuity. We present the following definition.

### Definition 5

Let $\mathcal{B}\in\mathcal{R}^{+}_{D(w)}$, $(a_{n})$ and $(b_{n})$ be sequences of non-negative integers. Also let $(u_{n})$ be a positive non-decreasing sequence. We say that a sequence $(x_{n})$ is deferred weighted $\mathcal{B}$-statistically convergent to a number *L* with the rate $o(u_{n})$ if, for every $\epsilon>0$,

$$\lim_{n\rightarrow\infty}\frac{1}{u_{n}P_{n}}\sum_{m=a_{n}+1}^{b_{n}} \sum_{k\in K_{\epsilon}}p_{m}b_{m,k}(i)=0\quad \textrm{uniformly in }i, $$

where

$$K_{\epsilon}=\bigl\{ k:k\leqq\mathbb{N}\mbox{ and } \vert x_{k}-L \vert \geqq\epsilon\bigr\} . $$

We write

$$x_{n}-L=\mathrm{stat}_{D(\bar{N})}^{\mathcal{B}}-o(u_{n}). $$

We now present and prove the following lemma.

### Lemma 1

*Let* $(u_{n})$ *and* $(v_{n})$ *be two positive non*-*decreasing sequences*. *Assume that* $\mathcal{B}\in\mathcal{R}^{+}_{D(w)}$, $(a_{n})$ *and* $(b_{n})$ *are sequences of non*-*negative integers*, *and let* $x=(x_{n})$ *and* $y=(y_{n})$ *be two sequences such that*

$$x_{n}-L_{1}=\mathrm{stat}_{D(\bar{N})}^{\mathcal{B}}-o(u_{n}) $$

*and*

$$y_{n}-L_{2}=\mathrm{stat}_{D(\bar{N})}^{\mathcal{B}}-o(v_{n}). $$

*Then each of the following assertions holds true*:

(i) $(x_{n}-L_{1})\pm(y_{n}-L_{2})=\mathrm{stat}_{D(\bar{N})}^{\mathcal{B}}-o(w_{n})$;
(ii) $(x_{n}-L_{1})(y_{n}-L_{2})=\mathrm{stat}_{D(\bar{N})}^{\mathcal{B}}-o(u_{n}v_{n})$;
(iii) $\gamma(x_{n}-L_{1})=\mathrm{stat}_{D(\bar{N})}^{\mathcal{B}}-o(u_{n})$ (*for any scalar* *γ*);
(iv) $\sqrt{ \vert x_{n}-L_{1} \vert }=\mathrm{stat}_{D(\bar{N})}^{\mathcal{B}}-o(u_{n})$,

*where* $w_{n}=\max\{u_{n}, v_{n}\}$.

### Proof

To prove assertion (i) of Lemma 1, we consider the following sets for $\epsilon>0$ and $x\in\mathcal{D}$:

$$\begin{aligned} &\mathcal{N}_{n}= \bigl\vert \bigl\{ k:k\leqq P_{n}\mbox{ and } \bigl\vert (x_{k}+y_{k} )-(L_{1}+L_{2}) \bigr\vert \geqq\epsilon \bigr\} \bigr\vert , \\ &\mathcal{N}_{0;n}= \biggl\vert \biggl\{ k:k\leqq P_{n} \mbox{ and } \vert x_{k}-L_{1} \vert \geqq \frac{\epsilon}{2} \biggr\} \biggr\vert , \end{aligned}$$

and

$$\mathcal{N}_{1,n}= \biggl\vert \biggl\{ k:k\leqq P_{n}\mbox{ and } \vert y_{k}-L_{2} \vert \geqq\frac{\epsilon}{2} \biggr\} \biggr\vert . $$

Clearly, we have

$$\mathcal{N}_{n}\subseteq \mathcal{N}_{0,n}\cup \mathcal{N}_{1,n} $$

which implies, for $n\in\mathbb{N}$, that

4.1 $$\begin{aligned} \lim_{n\rightarrow\infty}\frac{1}{P_{n}}\sum _{m=a_{n}+1}^{b_{n}}\sum_{k\in\mathcal{N}_{n}}p_{m}b_{m,k}(i) \leqq& \lim_{n\rightarrow\infty}\frac{1}{P_{n}}\sum _{m=a_{n}+1}^{b_{n}}\sum_{k\in\mathcal{N}_{0,n}}p_{m}b_{m,k}(i) \\ &{}+\lim_{n\rightarrow\infty}\frac{1}{P_{n}}\sum _{m=a_{n}+1}^{b_{n}}\sum_{k\in\mathcal{N}_{1,n}}p_{m}b_{m,k}(i). \end{aligned}$$

Moreover, since

4.2 $$ w_{n}=\max\{u_{n}, v_{n}\}, $$

by (4.1) we get

4.3 $$\begin{aligned} \lim_{n\rightarrow\infty}\frac{1}{w_{n}P_{n}}\sum _{m=a_{n}+1}^{b_{n}}\sum_{k\in\mathcal{N}_{n}}p_{m}b_{m,k}(i) \leqq& \lim_{n\rightarrow\infty}\frac{1}{u_{n}P_{n}}\sum _{m=a_{n}+1}^{b_{n}}\sum_{k\in\mathcal{N}_{0,n}}p_{m}b_{m,k}(i) \\ &{}+\lim_{n\rightarrow\infty}\frac{1}{v_{n}P_{n}}\sum _{m=a_{n}+1}^{b_{n}}\sum_{k\in\mathcal{N}_{1,n}}p_{m}b_{m,k}(i). \end{aligned}$$

Also, by applying Theorem 3, we obtain

4.4 $$ \lim_{n\rightarrow\infty}\frac{1}{w_{n}P_{n}} \sum _{m=a_{n}+1}^{b_{n}}\sum_{k\in\mathcal{N}_{n}}p_{m}b_{m,k}(i)=0 \quad \textrm{uniformly in } i. $$

Thus, assertion (i) of Lemma 1 is proved.

As assertions (ii) to (iv) of Lemma 1 are quite similar to (i), so it can be proved along similar lines. Hence, the proof of Lemma 1 is completed. □

We remind the modulus of continuity of a function of two variables $f(x,y)\in C_{B}(\mathcal{D})$ as

4.5 $$ \omega(f;\delta)=\sup_{(s,t),(x,y)\in \mathcal{D}} \bigl\{ \bigl\vert f(s,t)-f(x,y) \bigr\vert : \sqrt{(s-x)^{2}+(t-y)^{2}}\leqq \delta \bigr\} \quad (\delta>0), $$

which implies

4.6 $$ \bigl\vert f(s,t)-f(x,y) \bigr\vert \leqq \omega \biggl[f; \sqrt{ \biggl(\frac{s}{1-s}-\frac{x}{1-x} \biggr)^{2}+ \biggl( \frac{t}{1-t}-\frac{y}{1-y} \biggr)^{2}} \biggr]. $$

Now we present a theorem to get the rates of deferred weighted $\mathcal{B}$-statistical convergence with the help of the modulus of continuity in (4.5).

### Theorem 4

*Let* $\mathcal{B}\in\mathcal{R}^{+}_{D(w)}$, *and* $(a_{n})$ *and* $(b_{n})$ *be sequences of non*-*negative integers*. *Let* $T_{n}:C_{B}(\mathcal{D})\rightarrow C_{B}(\mathcal{D})$ *be sequences of positive linear operators*. *Also let* $(u_{n})$ *and* $(v_{n})$ *be positive non*-*decreasing sequences*. *We assume that the following conditions* (i) *and* (ii) *are satisfied*:

(i) $\Vert T_{n}(1;x,y)-1 \Vert _{C_{B}(\mathcal{D})}=\mathrm{stat}_{D(\bar{N})}^{\mathcal{B}}-o(u_{n})$;
(ii) $\omega(f,\lambda_{n})=\mathrm{stat}_{D(\bar{N})}^{\mathcal{B}}-o(v_{n})$ *on* $\mathcal{D}$,

*where*

$$\lambda_{n}=\sqrt{ \bigl\Vert T_{n}\bigl( \varphi^{2}(s,t),x,y\bigr) \bigr\Vert _{C_{B}(\mathcal{D})}} \quad \textrm{with }\varphi(s,t)= \biggl(\frac{s}{1-s}-\frac{x}{1-x} \biggr)^{2}+ \biggl(\frac{t}{1-t}-\frac{y}{1-y} \biggr)^{2}. $$

*Then*, *for every* $f\in C_{B}(\mathcal{D})$, *the following assertion holds true*:

4.7 $$ \bigl\Vert T_{n}\bigl(f(s,t);x,y\bigr)-f(x,y) \bigr\Vert _{C_{B}(\mathcal{D})}=\mathrm{stat}_{D(\bar{N})}^{\mathcal{B}}-o(w_{n}), $$

*where* $(w_{n})$ *is given by* (4.2).

### Proof

Let $f\in C_{B}(\mathcal{D})$ and $(x,y)\in \mathcal{D}$. Using (4.6), we have

$$\begin{aligned} \bigl\vert T_{n}(f;x,y)-f(x,y) \bigr\vert \leqq& T_{n} \bigl( \bigl\vert f(s,t)-f(x,y) \bigr\vert ;x,y\bigr)+ \bigl\vert f(x,y) \bigr\vert \bigl\vert T_{n}(1;x,y)-1 \bigr\vert \\ \leqq& T_{n} \biggl(\frac{\sqrt{ (\frac{s}{1-s}-\frac{x}{1-x} )^{2} + (\frac{t}{1-t}-\frac{y}{1-y} )^{2}}}{\delta}+1;x,y \biggr)\omega(f,\delta) \\ &{}+N \bigl\vert T_{n}(1;x,y)-1 \bigr\vert \\ \leqq& \biggl(T_{n}(1;x,y)+\frac{1}{\delta^{2}}T_{n}\bigl( \varphi(s,t);x,y\bigr) \biggr)\omega(f,\delta)+N \bigl\vert T_{n}(1;x,y)-1 \bigr\vert , \end{aligned}$$

where

$$N= \Vert f \Vert _{C_{B}(\mathcal{D})}. $$

Taking the supremum over $(x,y)\in\mathcal{D}$ on both sides, we have

$$\begin{aligned} &\bigl\Vert T_{n}(f;x,y)-f(x,y) \bigr\Vert _{C_{B}(\mathcal{D})} \\ &\quad \leqq \omega(f,\delta) \biggl\{ \frac{1}{\delta^{2}} \bigl\Vert T_{n}\bigl( \varphi(s,t);x,y\bigr) \bigr\Vert _{C_{B}(\mathcal{D})}+ \bigl\Vert T_{n}(1;x,y)-1 \bigr\Vert _{C_{B}(\mathcal{D})}+1 \biggr\} \\ &\qquad {}+N \bigl\Vert T_{n}(1;x,y)-1 \bigr\Vert _{C_{B}(\mathcal{D})}. \end{aligned}$$

Now, putting $\delta=\lambda_{n}=\sqrt{T_{n}(\varphi^{2};x,y)}$, we get

$$\begin{aligned} &\bigl\Vert T_{n}(f;x,y)-f(x,y) \bigr\Vert _{C_{B}(\mathcal{D})} \\ &\quad \leqq \omega(f,\lambda_{n}) \bigl\{ \bigl\Vert T_{n}(1;x,y)-1 \bigr\Vert _{C_{B}(\mathcal{D})}+2 \bigr\} +N \bigl\Vert T_{n}(1;x,y)-1 \bigr\Vert _{C_{B}(\mathcal{D})} \\ &\quad \leqq \omega(f,\lambda_{n}) \bigl\Vert T_{n}(1;x,y)-1 \bigr\Vert _{C_{B}(\mathcal{D})}+2\omega(f,\lambda_{n})+N \bigl\Vert T_{n}(1;x,y)-1 \bigr\Vert _{C_{B}(\mathcal{D})}. \end{aligned}$$

So, we have

$$\begin{aligned} & \bigl\Vert T_{n}(f;x,y)-f(x,y) \bigr\Vert _{C_{B}(\mathcal{D})} \\ &\quad \leqq\mu \bigl\{ \omega(f,\lambda_{n}) \bigl\Vert T_{n}(1;x,y)-1 \bigr\Vert _{C_{B}(\mathcal{D})}+\omega(f, \lambda_{n})+ \bigl\Vert T_{n}(1;x,y)-1 \bigr\Vert _{C_{B}(\mathcal{D})} \bigr\} , \end{aligned}$$

where

$$\mu=\max\{2,N\}. $$

For given $\epsilon>0$, we consider the following sets:

4.8 $$\begin{aligned} \mathcal{H}_{n} =& \bigl\{ n:n\leqq P_{n} \textrm{ and } \bigl\Vert T_{n}(f;x,y)-f(x,y) \bigr\Vert _{C_{B}(\mathcal{D})}\geqq\epsilon \bigr\} ; \end{aligned}$$

4.9 $$\begin{aligned} \mathcal{H}_{0,n} =& \biggl\{ n:n\leqq P_{n} \textrm{ and }\omega(f,\lambda_{n}) \bigl\Vert T_{n}(f;x,y)-f(x,y) \bigr\Vert _{C_{B}(\mathcal{D})}\geqq\frac{\epsilon}{3\mu} \biggr\} ; \end{aligned}$$

4.10 $$\begin{aligned} \mathcal{H}_{1,n} =& \biggl\{ n:n\leqq P_{n} \textrm{ and }\omega(f,\lambda_{n})\geqq\frac{\epsilon}{3\mu} \biggr\} \end{aligned}$$

and

4.11 $$ \mathcal{H}_{2,n}= \biggl\{ n:n\leqq P_{n} \textrm{ and } \bigl\Vert T_{n}(1;x,y)-1 \bigr\Vert _{C_{B}(\mathcal{D})} \geqq\frac{\epsilon}{3\mu} \biggr\} . $$

Lastly, for the sake of conditions (i) and (ii) of Theorem 3 in conjunction with Lemma 1, inequalities (4.8)–(4.11) lead us to assertion (4.7) of Theorem 4.

This completes the proof of Theorem 4. □

## Concluding remarks and observations

In this concluding section of our investigation, we present several further remarks and observations concerning various results which we have proved here.

### Remark 3

Let $(x_{n})_{n\in \mathbb{N}}$ be the sequence given in Example 2. Then, since

$$\mathrm{stat}_{D(\bar{N})}\lim_{n\rightarrow\infty}x_{n} \rightarrow\frac{1}{2} \quad \textrm{on }C_{\mathcal{B}}(\mathcal{D}), $$

we have

5.1 $$ \mathrm{stat}_{D(\bar{N})}\lim_{n\rightarrow\infty} \bigl\Vert T_{n}(f_{j};x,y)-f_{j}(x,y) \bigr\Vert _{C_{\mathcal{B}}(\mathcal{D})}=0 \quad (j=0,1,2,3). $$

Therefore, by applying Theorem 3, we write

5.2 $$ \mathrm{stat}_{D(\bar{N})}\lim_{n\rightarrow\infty} \bigl\Vert T_{n}(f;x,y)-f(x,y) \bigr\Vert _{C_{\mathcal{B}}(\mathcal{D})}=0, \quad f\in C_{\mathcal{B}}(\mathcal{D}), $$

where

$$\begin{aligned} f_{0}(s,t)&=1,\qquad f_{1}(s,t)=\frac{s}{1-s},\qquad f_{2}(s,t)=\frac{t}{1-t}\quad \textrm{and}\\ f_{3}(s,t)&= \biggl(\frac{s}{1-s} \biggr)^{2}+ \biggl( \frac{t}{1-t} \biggr)^{2}. \end{aligned}$$

However, since $(x_{n})$ is not ordinarily convergent, it does not converge uniformly in the ordinary sense. Thus, for the operators defined in (3.10) the traditional Korovkin-type theorem does not work. Hence, this application clearly indicates that our Theorem 3 non-trivially generalizes (is stronger than) the usual Korovkin-type theorem (see [32]).

### Remark 4

Let $(x_{n})_{n\in \mathbb{N}}$ be the sequence as given in Example 2. Then

$$\mathrm{stat}_{D(\bar{N})}\lim_{n\rightarrow\infty} x_{n} \rightarrow\frac{1}{2}\quad \textrm{on } C_{\mathcal{B}}(\mathcal{D}), $$

so (5.1) holds. Now, by applying (5.1) and our Theorem 3, condition (5.2) holds. However, since $(x_{n})$ is not statistically weighted $\mathcal{B}$-summable, so we can demand that the result of Kadak *et al.* [1], p. 85, Theorem 3, does not hold true for our operator defined in (3.10). Thus, our Theorem 3 is also a non-trivial extension of Kadak *et al.* [1], p. 85, Theorem 3, and [21]. Based upon the above results, it is concluded here that our proposed method has successfully worked for the operators defined in (3.10), and therefore it is stronger than the ordinary and statistical versions of the Korovkin-type approximation theorem (see [1, 32], and [21]) established earlier.

### Remark 5

We replace conditions (i) and (ii) in our Theorem 4 by the condition

5.3 $$ \bigl\vert T_{n}(f_{j};x,y)-f_{j}(x,y) \bigr\vert _{C_{\mathcal{B}}(\mathcal{D})}= \mathrm{stat}_{D(\bar{N})}^{\mathcal{B}}-o(u_{n_{j}}) \quad (j=0,1,2,3). $$

Now, we can write

5.4 $$ T_{n}\bigl(\varphi^{2};x,y\bigr)=\mathcal{F} \sum_{j=0}^{3} \bigl\Vert T_{n} \bigl(f_{j}(s,t);x,y\bigr)-f_{j}(x,y) \bigr\Vert _{C_{B}(\mathcal{D})}, $$

where

$$\mathcal{F}= \biggl\{ \epsilon+M+\frac{4M}{\delta^{2}} \biggr\} ,\quad (j=0,1,2,3). $$

It now follows from (5.3), (5.4), and Lemma 1 that

5.5 $$ \lambda_{n}=\sqrt{T_{n}\bigl( \varphi^{2}\bigr)}=\mathrm{stat}_{D(\bar{N})}^{\mathcal{B}}-o(d_{n}) \quad \textrm{on }C_{\mathcal{B}}(\mathcal{D}), $$

where

$$o(d_{n})=\max\{u_{n_{0}},u_{n_{1}},u_{n_{2}},u_{n_{3}} \}. $$

Thus, we fairly obtain

$$\omega(f,\delta)=\mathrm{stat}_{D(\bar{N})}^{\mathcal{B}}-o(d_{n}) \quad \textrm{on } C_{\mathcal{B}}(\mathcal{D}). $$

By using (5.5) in Theorem 4, we certainly obtain, for all $f\in C_{\mathcal{B}}(\mathcal{D})$, that

5.6 $$ T_{n}(f;x,y)-f(x,y)=\mathrm{stat}_{D(\bar{N})}^{\mathcal{B}}-o(d_{n}) \quad \textrm{on }C_{\mathcal{B}}(\mathcal{D}). $$

Therefore, if we use condition (5.3) in Theorem 4 instead of conditions (i) and (ii), then we obtain the rates of statistical deferred weighted $\mathcal{B}$-summability of the sequence $(T_{n})$ of positive linear operators in Theorem 3.
